# Supplementary material for: Analysis of the specificity of a COVID-19 antigen test in the Slovak mass testing program
Source: PLoS One. 2021 Jul 29;16(7):e0255267. doi: 10.1371/journal.pone.0255267 (PMC8320988; doi:10.1371/journal.pone.0255267)
Supplement: S1 Table — Counties are ordered from low to high incidence by the upper confidence bound. Data source [11]. (PDF) [file pone.0255267.s001.pdf]

S1 Table

County	Number of tests	Number of positive Tests	Positivity rate	Lower bound	Upper bound	Standard error
Rožňava	34307	100	0.29 %	0.20 %	0.40 %	0.03 %
Revúca	21419	58	0.27 %	0.17 %	0.41 %	0.04 %
Bratislava IV	65861	229	0.35 %	0.27 %	0.43 %	0.02 %
Bratislava III	49788	175	0.35 %	0.27 %	0.45 %	0.03 %
Veľký Krtíš	24282	76	0.31 %	0.20 %	0.46 %	0.04 %
Bratislava V	66135	263	0.40 %	0.32 %	0.49 %	0.02 %
Banská Štiavnica	11695	33	0.28 %	0.14 %	0.49 %	0.05 %
Bratislava I	28614	103	0.36 %	0.25 %	0.50 %	0.04 %
Bratislava II	80958	345	0.43 %	0.35 %	0.51 %	0.02 %
Rimavská Sobota	46872	196	0.42 %	0.32 %	0.53 %	0.03 %
Galanta	64841	303	0.47 %	0.38 %	0.57 %	0.03 %
Žiar nad Hronom	25816	108	0.42 %	0.29 %	0.57 %	0.04 %
Senec	71566	351	0.49 %	0.41 %	0.59 %	0.03 %
Piešťany	39080	185	0.47 %	0.36 %	0.60 %	0.03 %
Košice III	11109	41	0.37 %	0.20 %	0.61 %	0.06 %
Levice	66411	340	0.51 %	0.42 %	0.61 %	0.03 %
Nové Zámky	83224	436	0.52 %	0.44 %	0.61 %	0.03 %
Malacky	54585	285	0.52 %	0.42 %	0.64 %	0.03 %
Pezinok	45801	240	0.52 %	0.42 %	0.65 %	0.03 %
Lučenec	40576	213	0.52 %	0.41 %	0.66 %	0.04 %
Košice IV	26992	135	0.50 %	0.37 %	0.66 %	0.04 %
Trebišov	61297	353	0.58 %	0.48 %	0.69 %	0.03 %
Košice - okolie	86759	524	0.60 %	0.52 %	0.70 %	0.03 %
Trnava	86220	532	0.62 %	0.53 %	0.71 %	0.03 %
Košice I	31728	187	0.59 %	0.45 %	0.75 %	0.04 %
Krupina	12975	66	0.51 %	0.32 %	0.76 %	0.06 %
Hlohovec	28475	171	0.60 %	0.46 %	0.77 %	0.05 %
Nitra	107411	738	0.69 %	0.60 %	0.78 %	0.03 %

County	Number of tests	Number of positive Tests	Positivity rate	Lower bound	Upper bound	Standard error
Zlaté Moravce	26056	156	0.60 %	0.45 %	0.78 %	0.05 %
Šaľa	31993	199	0.62 %	0.48 %	0.79 %	0.04 %
Komárno	66187	457	0.69 %	0.59 %	0.81 %	0.03 %
Poltár	12455	71	0.57 %	0.37 %	0.84 %	0.07 %
Zvolen	38254	274	0.72 %	0.58 %	0.88 %	0.04 %
Žarnovica	15918	105	0.66 %	0.46 %	0.91 %	0.06 %
Košice II	39314	295	0.75 %	0.61 %	0.91 %	0.04 %
Gelnica	17755	129	0.73 %	0.53 %	0.97 %	0.06 %
Michalovce	58929	512	0.87 %	0.74 %	1.01 %	0.04 %
Myjava	17753	139	0.78 %	0.58 %	1.04 %	0.07 %
Nové Mesto nad Váhom	40829	363	0.89 %	0.74 %	1.06 %	0.05 %
Dunajská Streda	87090	840	0.96 %	0.86 %	1.08 %	0.03 %
Senica	40675	384	0.94 %	0.79 %	1.12 %	0.05 %
Prešov	99798	1031	1.03 %	0.93 %	1.15 %	0.03 %
Banská Bystrica	62169	669	1.08 %	0.94 %	1.22 %	0.04 %
Vranov nad Topľou	43552	460	1.06 %	0.90 %	1.23 %	0.05 %
Detva	19629	211	1.07 %	0.84 %	1.35 %	0.07 %
Turčianske Teplice	11287	112	0.99 %	0.70 %	1.35 %	0.09 %
Žilina	103957	1288	1.24 %	1.13 %	1.36 %	0.03 %
Trenčín	73424	894	1.22 %	1.08 %	1.36 %	0.04 %
Ilava	37604	442	1.18 %	0.99 %	1.38 %	0.06 %
Sobrance	12986	135	1.04 %	0.76 %	1.38 %	0.09 %
Brezno	36779	447	1.22 %	1.03 %	1.42 %	0.06 %
Skalica	29223	368	1.26 %	1.05 %	1.50 %	0.07 %
Martin	56533	771	1.36 %	1.20 %	1.54 %	0.05 %
Spišská Nová Ves	53976	738	1.37 %	1.20 %	1.55 %	0.05 %
Považská Bystrica	37822	505	1.34 %	1.14 %	1.55 %	0.06 %
Stropkov	10494	125	1.19 %	0.86 %	1.60 %	0.11 %
Liptovský Mikuláš	45606	640	1.40 %	1.22 %	1.60 %	0.06 %
Svidník	16631	220	1.32 %	1.04 %	1.65 %	0.09 %
Dolný Kubín	24029	345	1.44 %	1.19 %	1.72 %	0.08 %

County	Number of tests	Number of positive Tests	Positivity rate	Lower bound	Upper bound	Standard error
Bytča	21059	311	1.48 %	1.21 %	1.78 %	0.08 %
Medzilaborce	6980	91	1.30 %	0.89 %	1.83 %	0.14 %
Topoľčany	45129	755	1.67 %	1.47 %	1.89 %	0.06 %
Bardejov	43562	728	1.67 %	1.47 %	1.89 %	0.06 %
Poprad	59072	1059	1.79 %	1.61 %	1.99 %	0.05 %
Námestovo	36661	670	1.83 %	1.60 %	2.08 %	0.07 %
Humenné	32632	598	1.83 %	1.59 %	2.10 %	0.07 %
Kežmarok	43959	845	1.92 %	1.71 %	2.16 %	0.07 %
Snina	19122	345	1.80 %	1.49 %	2.16 %	0.10 %
Partizánske	26359	490	1.86 %	1.59 %	2.16 %	0.08 %
Ružomberok	33048	626	1.89 %	1.65 %	2.16 %	0.07 %
Kysucké Nové Mesto	20605	383	1.86 %	1.55 %	2.20 %	0.09 %
Prievidza	77918	1596	2.05 %	1.88 %	2.23 %	0.05 %
Bánovce nad Bebravou	23128	455	1.97 %	1.67 %	2.30 %	0.09 %
Levoča	18344	373	2.03 %	1.70 %	2.41 %	0.10 %
Sabinov	35366	804	2.27 %	2.01 %	2.56 %	0.08 %
Tvrdošín	20200	448	2.22 %	1.88 %	2.59 %	0.10 %
Stará Ľubovňa	28749	805	2.80 %	2.48 %	3.15 %	0.10 %
Púchov	29455	830	2.82 %	2.50 %	3.16 %	0.10 %
Čadca	53907	1691	3.14 %	2.89 %	3.40 %	0.08 %

S1 Table: County, number of tests, number of positive tests, positivity rates, simultaneous Bonferroni adjusted 95% confidence intervals, and standard errors of the positivity rates in the 79 counties. Counties are ordered from low to high incidence by the upper confidence bound. Data source [11].
