# Supplementary material for: Analysis of the specificity of a COVID-19 antigen test in the Slovak mass testing program
Source: PLoS One. 2021 Jul 29;16(7):e0255267. doi: 10.1371/journal.pone.0255267 (PMC8320988; doi:10.1371/journal.pone.0255267)
Supplement: Attachment — Submitted filename: Point to Point reply.pdf [file pone.0255267.s002.pdf]

We thank the referee for the helpful remarks and revised the manuscript accordingly. In addition we formatted the document according to the PLOS ONE formatting guidelines. Please see below a point to point answer to the issues raised.

Point to Point reply to the Referee's Reports

Reviewer #1: In presenting argument in the introduction that antibody testing can be an alternative for RT-CPR, it may be helpful to note that the test evaluates different things: active infection vs history of infection (including current), with different delays between tests being positive (longer for serology). This reviewer agrees that serology can be a very attractive alternative for reasons cited by the authors, but additional caveats other than SN (sensitivity) and SP (specificity) related to meaning of the test in terms of inference of onset of incident infection should be clearly presented.

In the introduction we refer to **antigen** (not antibody) testing. Antigen tests, like PCR tests, are used to detect active infections. They deliver test results quicker than PCR tests and are more economic and are therefore used in mass testing programs.

Can you please be clearer in the introduction how your approach is "in contrast to other studies which use a PCR-test as a reference to estimate sensitivity and specificity of an Ag-Test4"?

We have added a sentence in the introduction to clarify how our approach differs:

"The lower bound for the specificity is obtained by making the conservative assumption that potentially all positive results are false positive results. This is in contrast to other studies which use a PCR-test as a reference to estimate sensitivity and specificity of an Ag-Test."

One alternative to Bonferroni correction that seems appealing to this reviewer is to model rate of positive tests using binomial regression with random effect of county, and maybe some fixed effects that can account for rate of positive tests, such as numbers quarantined per county, positivity rates from Phase 1, age structure, economic indicators, percent of county tested, outdoor temperature, count/proportion of outdoor test sites, date of test (the usual confounders in epidemiology that can affect both willingness to test and chance of having been infected plus those mentioned in the discussion by the authors). The fixed intercept of the model would overall rate of positive tests and can be made county-specific through use of other random and fixed covariates; upper percentile of these modelled estimates can be used authors did with their Bonferroni-adjusted confidence interval. One may be also tempted to model spatial correlation, if descriptive statistics support its existence.

We agree that the data can be analyzed with more complex models, using a joint model for all countries and taking other data sources into account (see e.g., Boďová, K. & Kollár 2020, or Pavelka, M. et al. 2021). However, the objective of this work is not to model incidence rates and explain their variation, but to arrive at a robust lower bound for the specificity of

the antigen test. For this objective, we believe that an analysis approach that relies on minimal assumptions and therefore is as robust as possible is more appropriate. Especially, our approach based on the Bonferroni correction and exact binomial confidence intervals is valid under all possible correlation structures between counties. Thus the validity of the derived lower bound does not rely on a specific spatial correlation model. The method gives robust estimates for the statistical uncertainty of the derived bound. Because of the large sample sizes, the obtained bound is still highly informative.

Instead of just giving upper bound of 95%CI, it would be more informative to give both the estimate per county and associated standard error. This should allow anyone to estimate different percentile if for their purposes 97.5% is not appropriate. This may already be in the supplemental materials, so maybe this is just a matter of pointing the reader in the right direction.

As suggested, we added a table with the data from all countries including the standard errors as supporting information and added a corresponding sentence in the caption of Figure 1.
